# Social encounter networks: characterizing Great Britain

**DOI:** 10.1098/rspb.2013.1037

**Published:** 2013-08-22

**Authors:** Leon Danon, Jonathan M. Read, Thomas A. House, Matthew C. Vernon, Matt J. Keeling

**Affiliations:** 1 Mathematics Institute, University of Warwick, Coventry CV4 7AL, UK; 2 School of Life Sciences, University of Warwick, Coventry CV4 7AL, UK; 3 Department of Epidemiology and Population Health, Institute of Infection and Global Health, University of Liverpool, Neston, CH64 7TE, UK; 4 University Computing Service, University of Cambridge, Cambridge CB2 3QH, UK

**Keywords:** social contact, survey, epidemic, infectious disease, network

## Abstract

A major goal of infectious disease epidemiology is to understand and predict the spread of infections within human populations, with the intention of better informing decisions regarding control and intervention. However, the development of fully mechanistic models of transmission requires a quantitative understanding of social interactions and collective properties of social networks. We performed a cross-sectional study of the social contacts on given days for more than 5000 respondents in England, Scotland and Wales, through postal and online survey methods. The survey was designed to elicit detailed and previously unreported measures of the immediate social network of participants relevant to infection spread. Here, we describe individual-level contact patterns, focusing on the range of heterogeneity observed and discuss the correlations between contact patterns and other socio-demographic factors. We find that the distribution of the number of contacts approximates a power-law distribution, but postulate that total contact time (which has a shorter-tailed distribution) is more epidemiologically relevant. We observe that children, public-sector and healthcare workers have the highest number of total contact hours and are therefore most likely to catch and transmit infectious disease. Our study also quantifies the transitive connections made between an individual's contacts (or clustering); this is a key structural characteristic of social networks with important implications for disease transmission and control efficacy. Respondents' networks exhibit high levels of clustering, which varies across social settings and increases with duration, frequency of contact and distance from home. Finally, we discuss the implications of these findings for the transmission and control of pathogens spread through close contact.

## 1. Introduction

The spread of respiratory infections within human communities and between populations is intimately related to the patterns of contacts made between individuals and the transmission opportunities presented by social interaction. While the network structure of such contacts is understood to have important implications for transmission and control of infections [1,2], there is a dearth of information about their structural form and how this varies between individuals and across cultural, geographical or social contexts.

The modelling of infection spread at the population scale has proved extremely useful for explaining observed patterns of disease prevalence, generating predictions and hence identifying optimal control strategies [3,4]. However, for a range of infectious diseases and potential control measures (e.g. contact-tracing), information about social mixing, contacts and related behaviours is required at the individual scale. A lack of detailed quantitative information has generally necessitated a range of simplifying assumptions regarding the structure of contact networks, such as power-law (or scale-free) distributions for the number of contacts and configuration models for generating connections between individuals. The type of assumptions made can have a profound impact on model predictions [2,5]. There is, therefore, an important need for empirical studies of social networks appropriate to infectious disease spread, to inform (or at least constrain) the types of contact networks that are realistic.

Heterogeneity in the number of social contacts has been identified as crucial to understanding infectious disease spread in populations [2–5]. Heavily right-skewed distributions of the number of contacts (as exemplified by core groups [6] or power-law distributions [7]) describe populations where most individuals have few contacts, but a small fraction of the population have many contacts. The disease dynamics arising from such forms of degree distributions have been the focus of a number of theoretical studies, with much emphasis on scale-free topologies [8,9]. There is, however, little empirical evidence that social contacts follow such patterns: previous large-scale contact diary studies may have constrained participant's recording of large numbers of contacts owing to study design issues, hampering a quantitative understanding of the extremes. Theoretical work with a variety of network types has demonstrated the sensitivity of basic epidemiological behaviour (such as early epidemic growth rates, final epidemic sizes and critical levels of vaccination) to the tail of the distribution of contacts [10]. Therefore, a detailed understanding of social contact distributions, particularly their right-hand tails (high number of contacts), is important for accurately understanding epidemiological dynamics.

An additional structural aspect of social contact networks is the clustering, or transitivity, of contacts. Clustering may be defined as the probability of contact occurring between the contacts of an individual or, from a network perspective, as the proportion of connected triples that form triangles [11]. Clustering of contacts has important implications for the speed at which infections can spread through a social network: increased clustering slows transmission for a given contact rate [11,12], whereas the efficacy of contact-tracing is improved by the presence of clustering [13,14]. Currently, few infection-orientated studies have measured clustering, despite its significance for disease dynamics and control. For self-reported contact diary studies, clustering has only previously been measured by re-constructing transitive links (triangles) between named contacts [15].

To improve the understanding of the character of social networks, we conducted an anonymized survey of the population of Great Britain (GB) through a postal- and web-based questionnaire to collect information on the types of social contact likely to lead to the transmission of infection. Our study design, the findings from this study and some implications for epidemiological understanding follow.

## 2. Methods

**Figure 1.**
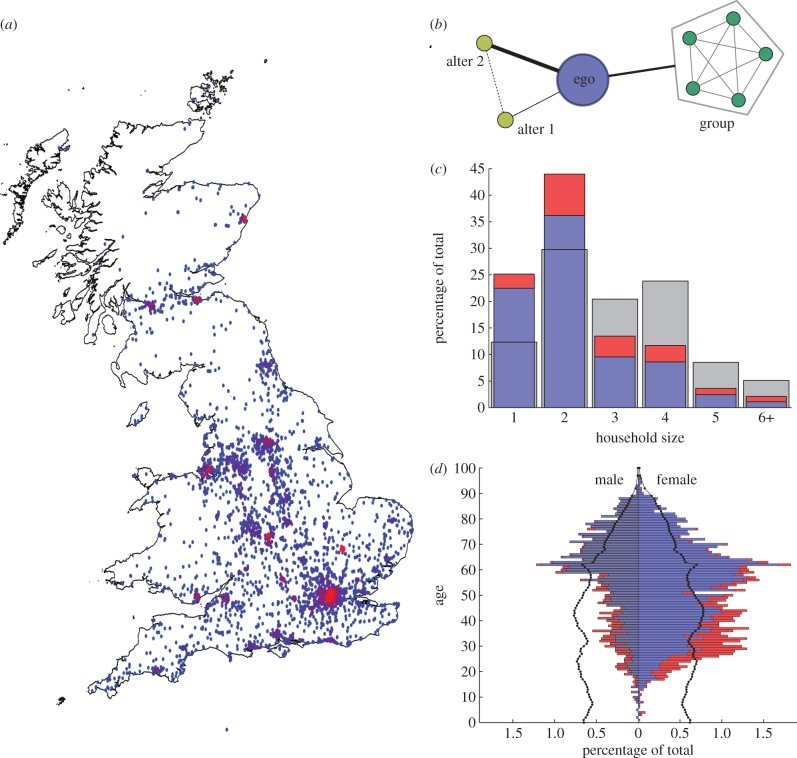
(*a*) Spatial distribution of respondents in the GB (4689 individuals provided a valid postcode); dots are colour-codes, so that regions of the highest density are in red, whereas low-density regions are in blue. There is good agreement between the location of respondents and major urban areas. (*b*) Example of an egocentric network collected by our survey. (*c*) Distribution of household sizes from the postal (blue) and online (red) surveys, compared with the national average (grey) showing that households of size 1 and 2 are over-represented. (*d*) Proportion of the respondents of a particular age and gender from the postal (blue) and online (red) surveys; the black lines show the estimated GB population percentages for 2009. These highlight the lack of young children and that males below 60 are under-represented.

We conducted a cross-sectional survey of households and individuals within GB, asking for self-reported information regarding social encounters made during a specified waking day. There were two recruitment arms to the study: a postal survey using a paper-based questionnaire sent to households in GB, and a web-based survey using an online questionnaire which was open for anyone to participate. The postal survey was distributed to randomly selected households within GB from the post office address list database, with a total of 140 000 posted during 2009. Information included within the postal survey packs directed other members of the household to the Internet-based survey. The study website http://www.contactsurvey.org hosting the survey was further promoted ad hoc via university press releases, social networking sites and other media outlets (local radio, local and national newspapers). In both cases, basic demographic data of participants were collected, including age and gender of respondent, the number of people in their household and the first part of their home postcode, providing an approximate location (figure 1*a*). The data collected by this study are available on Warwick Research Archive Project at http://wrap.warwick.ac.uk/54273/.

The postal questionnaire (see electronic supplementary material, appendix) was designed to fit on a single side of an A3 sheet, and was colour-coded for visual impact and ease of completion. Detailed instructions and an introduction to the science behind the questionnaire were also included. To aid completion, participants could write the names (or nicknames) of contacts and groups on the left-hand side of the questionnaire; participants were advised to detach this section before returning the results, thereby preserving the anonymity of all parties. Instructions requested a single member of the household to complete the survey, with the suggestion that this could be the person in the household with the most recent birthday.

Questionnaires asked about all contacts a participant met on a given day (defined as from waking to sleeping). A contact was defined in the same way as a number of previous studies [15,16]: a person with whom the participant had had a face-to-face conversation (within 3 m) and/or skin-on-skin physical touch. To facilitate the reporting of large numbers of contacts, questionnaires permitted participants to record groups of similar contacts. This could either refer to a group of individuals that were all met simultaneously (e.g. a business meeting of 20 people) or to a large number of individuals that were met separately under similar circumstances (e.g. serving lots of different customers). With postal questionnaires, participants could record up to 20 individual contacts, up to five groups of contacts (of up to 999 individuals within a group), and up to 999 extra contacts about which no other information was collected (only 46 respondents used this additional box). Online questionnaires had no design restriction on the number of individual contacts or group contacts, and detailed information was asked for all of these.

For each contact, we asked of the participant: (i) if their contact involved skin-on-skin touch; (ii) the settings in which the contact was encountered during the day; (iii) the distance from home where encounters with that person took place; (iv) the total time spent with that person during the day; (v) how often the participant would expect to meet that person (see electronic supplementary material, for categories). In the case of groups, contact characteristics reported were assumed to apply to every individual in the group, with the exception of contact time. In accounting for group contact time, we place a strict upper limit of 20 h on the total contact time with a group, because we deem it impossible to have close face-to-face conversations with every member of a group of 20 people for over an hour each. In such cases, we assume that the respondent has incorrectly interpreted our instructions (i.e. they met a group of 20 people, and in total, the meeting lasted over an hour). In these cases (502 out of a total of 4642), we rescale the time by dividing the estimated total time by the number in the group to get an individual value. When the total contact duration is less than 20 h, whether the instructions have been interpreted correctly is determined probabilistically to match the general profile of contact times.

A novel aspect of our survey was asking each respondent to inform us whether they believed pairs of contacts had met each other in the past week, thus forming a transitive link between these contacts. Both postal and online surveys sought to measure transitive links, or clustering of contacts. For the postal survey, participants were asked which of their individual contacts were thought to have met each other during the reported day or in the previous week. To obtain a measure for groups in the postal survey, participants were asked whether most of the people within the group met each other (‘yes/no’). Owing to space constraints in the postal survey, transitive contacts between pairs of groups and groups and individuals were not collected. The greater flexibility offered by the online survey allowed participants to report the transitive encounters between all combinations of individual and group contacts.

We use this information to calculate individual-level clustering for each respondent. In the simplest (unweighted) measure, we define clustering as the proportion of contact pairs around an individual that are believed to have met each other in the past week. To account for the difference between online and postal surveys, we normalize the clustering coefficient by the maximum number of transitive links it was possible to capture by the survey method (see electronic supplementary material, for details). This calculation is made more complex by groups of individuals, where we ask whether most of the group met another contact; as a minimal approximation, we assume that only half of the group takes part in such transitive contacts.

A secondary issue is that transitive links are reported for a 7-day period, whereas contacts are reported for a single day. While this distinction is important for a rigorous definition of clustering within the network, the 7-day timescale may be considered more useful from an epidemiological perspective as a means of identifying multiple transmission routes. Analysis of data from a previous study [15] suggests that aggregation of transitive links over 7 days increases the estimated clustering values by a factor of 1.8 (see electronic supplementary material).

Throughout this paper, we actually consider a slightly more involved measure of clustering, where the pairs of contacts are weighted by their associated contact durations (please see electronic supplementary material for details on weighting). This gives a more natural measure as it gives more emphasis to long duration and therefore more epidemiologically important contacts. When this weighted clustering is close to one, it indicates that the majority of longer duration contacts are estimated to have met each other. Such high clustering leads to a reduction in the spread of infection owing to local competition for susceptible individuals [14].

All confidence intervals reported are measured by bootstrapping from the data, and considering the interval containing 95 per cent of the values.

## 3. Results

The postal survey generated 3901 responses, yielding an overall response rate of around 2.7 per cent; the public willingness to participate was probably influenced by heightened awareness of the ongoing influenza A/H1N1pdm09 pandemic. The online survey generated 1126 responses from residents of GB to the end of September 2010. In total, we collected a total of 134 484 contacts from 5027 GB participants across the postal- and web-based surveys, of which 40 462 were individual contacts and 4642 were groups with a variety of sizes. The results from each participant were used to generate an egocentric network—a localized network detailing the contacts of the respondent and the links between these contacts (see figure 1*b* for a stylized example).

The responses received show a sample of the GB population which was only partly representative. While there was generally good spatial coverage matching high-density populations (figure 1*a*), there are consistent biases in the age, gender and household composition of respondents (figure 1*c,d*). This is to be expected and is in keeping with the general results of other surveys and questionnaires dealing with health issues [17,18]. In general, females (66% of respondents; figure 1*d*) were more likely to appear in our sample than males (34% of respondents). For males, those over 60 years old (and less than 90) were more likely to appear in our sample, compared with the population distribution (shown as a black line); for women, almost all ages between 25 and 80 years old are over-represented. In addition, there is a clear demographic difference between those that complete the web survey compared with the postal one; a much younger set of respondents used the online questionnaire, with an average age of 37 compared with 56. We note that few respondents (less than 1% of the total) were 16 years old or under, which makes assessment of the mixing behaviour of pre-school and school-age children difficult. As such, our findings are most informative about the social contact network for the adult population of GB. We found little bias in postal response rates for different survey days of the week (see the electronic supplementary material,  table S1). Finally, we observe that one- and two-person households are over-represented and therefore we may not fully capture all aspects of strong, within-household contacts.

### (a) Numbers of contacts and total contact time

**Figure 2.**
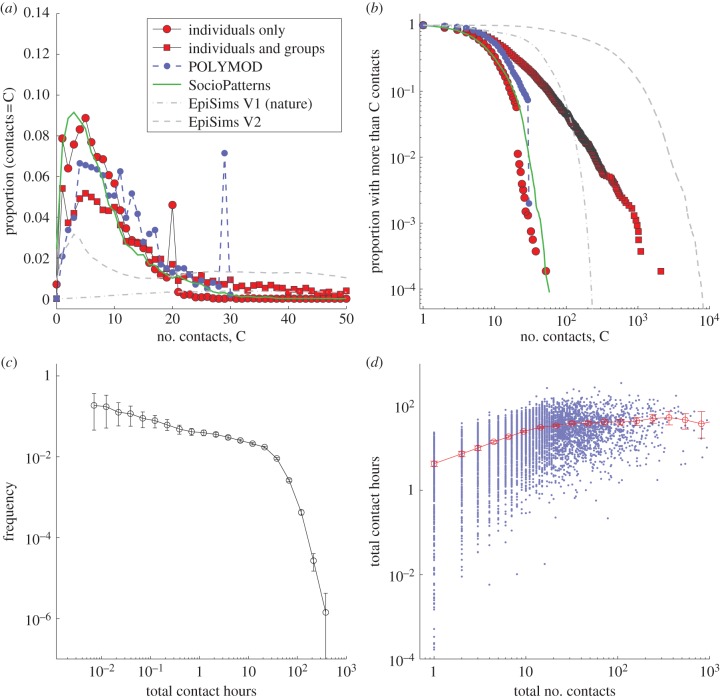
Distributions of the number of contacts and total contact time per individual. (*a*,*b*) The correspondence between distributions from our survey distributions and distributions from other estimates of human contacts—in particular, the POLYMOD study [16], the SocioPatterns study [19] and the EpiSims model [20]. In (*a*), we show the frequency of respondents with relatively low numbers of contacts, whereas in (*b*), we plot the cumulative frequency on a logarithmic scale to provide a clearer visual representation and highlighting the tail of the distribution. (*c*) The distribution of the total contact time on a logarithmic scale, with the error bars showing the confidence intervals from 1000 bootstrapped samples, and (*d*) shows the relationship between the total contact time and the number of contacts, the blue points showing results for each respondent and the red line showing the mean values and confidence intervals from 1000 bootstrap samples.

The local structure of the respondents' ego networks is high-dimensional and therefore cannot be comprehensively captured by any single quantity. However, two measures provide important, epidemiologically relevant characterizations of local networks: the number of contacts and the total contact time (figure 2). The number of contacts allows us to quantify the importance of an individual within the population-level network, and quantifies the transmission potential from this individual for a highly transmissible infection (such as norovirus or some haemorrhagic fevers) when the duration of contact can be neglected. By contrast, the transmission of many infections is limited by the duration of contact, in which case the total contact time (the sum of the times for all contacts) provides a more appropriate indicator of risk for both infection and transmission. However, both of these measures also have limitations: the number of contacts does not differentiate between long- and short-duration encounters, whereas the total contact time cannot distinguish between many short-duration and few long-duration encounters. In addition, neither measure can account for the epidemiological consequences of heterogeneity in the intimacy of the contacts nor the implications of structure within the local contact network. Throughout this paper, we focus on understanding the distribution and heterogeneity of these two measures, but first we consider how contacts are recorded in our survey as either individuals or groups.

The mean number of individual contacts recorded by respondents is 7.97, whereas the mean total number of contacts (including those in groups) is 26.75. These averages rise slightly to 8.28 and 28.50, when we correct for age and gender biases in our sample (compared with the population). However, these mean values do not convey the considerable heterogeneity in the numbers of contacts that respondents reported; figure 2 highlights this heterogeneity. Figure 2*a* shows the proportion of respondents with a given number of contacts (or degree, *k*) and focuses on the bulk, most commonly reported contact numbers. By contrast, figure 2*b* shows the proportion of respondents with at least a particular number of contacts, and uses a logarithmic scale to enable the full spectrum of contact numbers to be shown. In both graphs, the number of individual contacts is shown with red circles, and the sum of individual and group contacts is plotted with red squares (the results of past studies are shown on the same axes for comparison).

The most frequent number of individual daily contacts are 1 and 5 (comprising nearly 8% and 9% of all respondents, respectively), although for the postal survey there is also a conspicuous peak at 20 (4.5% of all respondents) which is the maximum number of individual contacts that could be recorded on the paper questionnaire. When the individual and group contacts are combined, the most frequently reported number of total contacts are again 1 and 5 (both at around 5% of all respondents), although the maximum number of total contacts reported rises to 3011. This distribution of total contacts is characterized by a lognormal body which captures the bulk of the distribution, together with a power-law tail (with an exponent of –2.45) which captures the distribution of high numbers of contacts [21].

Three other main published studies (shown in figure 2*a*,*b*) have previously attempted to quantify such social contact patterns: EpiSims [20]; POLYMOD [16] and SocioPatterns [19]. The observed power-law tail in our results has clear resonances with previous studies of synthetic populations [20], whereas the bulk properties more closely match the findings of direct measurements [16,19]. Both our count of individual contacts and the POLYMOD study are limited by the number of contacts that can be listed on the questionnaire (20 in our study and 30 in POLYMOD) which produces a clear frequency peak at the maximum (figure 2*a*). Our use of groups helps to alleviate this issue producing a smoother distribution. Despite these issues, there is relatively good agreement between POLYMOD and our survey for those with low numbers of contacts; having between 4 and 7 contacts per day is relatively common in both studies. However, in our survey, we also have a significant number of respondents with either very low or very large number of contacts, which is in far closer agreement with the theoretical networks of EpiSims. Finally, we note the striking agreement between our individual results and the findings of SocioPatterns [19] where contacts between individuals were recorded using RFID tags.

Attention is now focused on the alternative epidemiological measure: total contact time (figure 2*c*,*d*). There is a strong correlation between the total number of contacts reported and the total contact time (figure 2*d*); this is unsurprising as having more contacts means that there is the potential to have more total interaction time. However, although short total contact times are possible, there are fewer large values (figure 2*c*). The frequency distribution is observed to have a heavy tail, but with a sharp decline at high values, implying that very long total contact times are exceedingly rare. Thus, the total contact time may be a more useful quantity to report, as aggregate properties will be less sensitive to extremes of behaviour.

For the rest of this paper, we focus on the total contact time for each respondent as we feel this provides a more natural measure and the better predictor of epidemiological risk for common infections.

### (b) Effect of age and occupation

**Figure 3.**
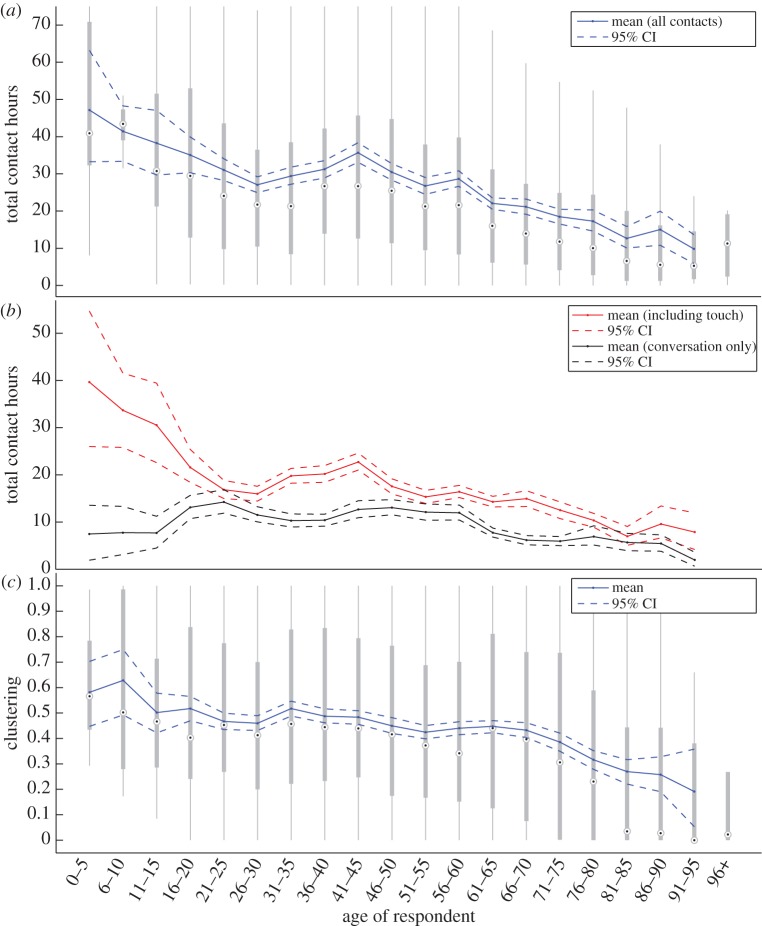
Relationship between age of respondent time, contact hours and clustering. (*a*) Box and whisker plots of the estimated total contact time, reported by age group. The black dots show median values, the grey boxes show the inter-quartile range and the whiskers extend to 1.5 times the size of the inter-quartile range from the quartiles; all points beyond that are considered to be outliers and are omitted for clarity. The blue line depicts the mean values together with the 95% confidence limits of those means derived from 1000 bootstrap samples for age ranges with more than five respondents. (*b*) The mean contact hours in each age group are partitioned into only those that involved touch (which may be considered more likely to pass infection; red) and those that are conversation only (black). (*c*) Box and whisker plots for the weighted clustering measured for each respondent.

The age of the respondent is known to be an important indicator of social mixing patterns and daily contacts [16]. Figure 3*a* shows the relationship between total contact hours and age of respondent. We see that school-age (5–15 years old) and pre-school (0–4 years) children are associated with the greatest contact times (as well as the greatest number of contacts, see electronic supplementary material). In general, total contact times decrease with age, although there is evidence of a slight increase in mid-30s to 40s, which we speculate is associated with either becoming parents of school-age children or related to work-based activities.

For many infections, very close contact may be required for transmission, therefore in figure 3*b*, we separate contacts into those involving touch and those that are conversation only. Conversational-only contacts dominate in terms of numbers of contacts, but contacts involving touch tend to be of longer duration and hence contribute more to the total contact time. Although displaying a similar pattern to figure 3*a*, the results for touch-based contacts show far greater heterogeneity with age, hence highlighting the role of children in the transmission of close-contact infections.

When considering clustering as a function of age, we again observe a striking age-dependent pattern (figure 3*c*), with children (0–10 years old) having very high clustering owing to the strong interactions within home, nursery or school groups. Clustering is maintained at around 0.5 for individuals aged 11–65 years old and then drops rapidly for older respondents.

**Figure 4.**
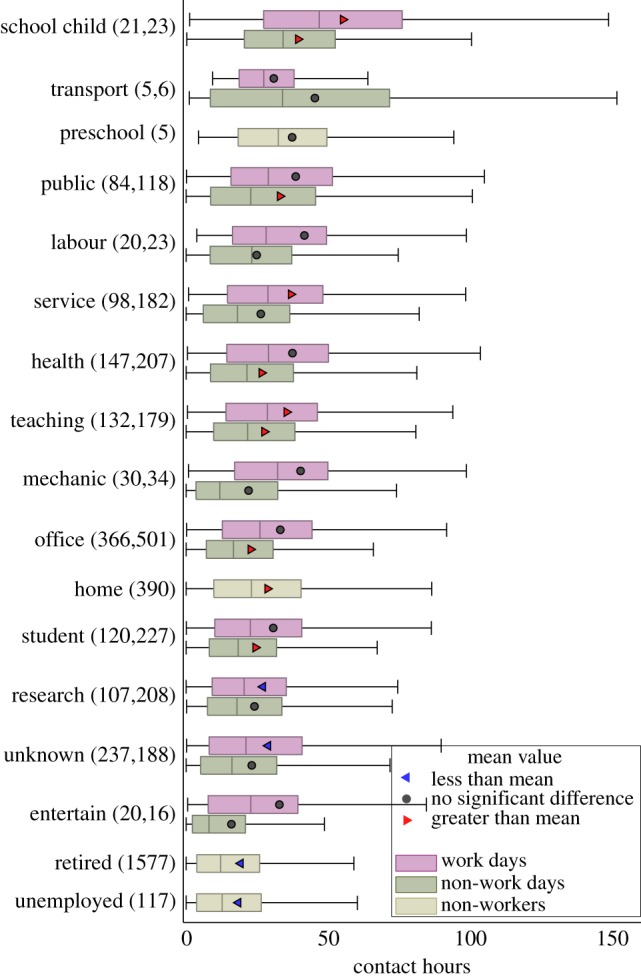
Heterogeneity in the number of contact hours by occupation. Box and whisker plot showing the median, quartiles and 95 percentiles of contact hours; occupations are ordered by median number of contact hours. For each occupation category, we show the number of respondents in brackets (work days and non-work days) and distinguish between days at work or school (red), from non-working days (green); for some occupations (pre-school, home, retired and unemployed, shown in grey), such a distinction is not possible. For each category, where the contact hour distributions are significantly greater or less than the total sampled population they are shown with a left-facing triangles or right-facing triangles symbol, respectively, and with an circles denotes when no significant difference is observed.

A second source of heterogeneity in contact patterns arises from a respondent's profession or occupation. Although the questionnaires allowed for a free response for occupation, for comparison, we categorized each occupation into one of a set of 17 basic classes (e.g. health, office or school child; see electronic supplementary material for more details). Of the 5027 respondents, 175 did not provide an occupation, whereas a further 200 could not be readily assigned to a particular class. Figure 4 shows the relationship between total contact hours and a participants occupation; where applicable, we separate results into those days when a respondent works (pink) and those when they do not (green).

Figure 4 reinforces our earlier findings that school children have predominantly more contact hours than the rest of the population, whereas retired (and therefore presumably older) people have substantially less. However, figure 4 allows us to delve into the impact of occupation in more detail; for example, teachers and service workers have significantly more contacts on working days than the national average, whereas researchers have significantly fewer (significance is established by Kolmogorov–Smirnov testing at the 95% level). In addition, unemployed people (although not individuals who have decided not to work, e.g. stay-at-home parents) tend to have the lowest number of contacts and are comparable with retired people. While such relationships between contact rate and occupations agree with intuition, our findings permit us to quantify these differences. For example, during a working day, a teacher or a healthcare worker, on average, has a least 50 per cent more contact hours than either unemployed or retired people.

### (c) Covariates of clustering and contact times

**Figure 5.**
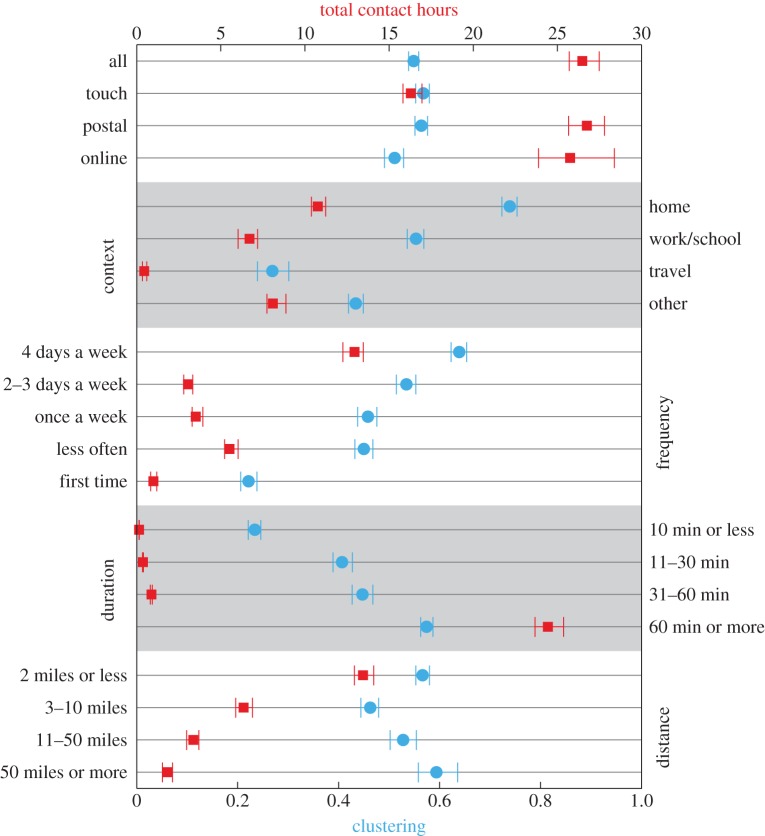
The variation in total contact hours and weighted clustering with other covariates measured in the survey. The total contact hours (red and top *x*-axis) depicts the average total time a respondent spends with contacts of a particular type. Weighted clustering of respondents ego networks (blue and lower *x*-axis), captures the proportion of transitive links between contacts of a given type. Confidence intervals are calculated by bootstrapping from the respondent sample and duration per contact.

The frequency with which contacts is encountered, the duration of those contacts and the distance travelled to meet those contacts all have important implications for the spread of infections [15]. Here, we examine how such elements influence the total contact time and the clustering of contacts (figure 5).

The average total contact time (red) across all respondents, postal and online surveys is between 25 and 27 h, showing that both methods generate comparable results. If we consider contacts who were touched only (and therefore potentially at a greater risk of infection), then the associated total contact time drops to 16 h. Considering the context in which the contacts occur, we find that home contacts (9% of contacts) account for the majority of the contact hours, whereas work (36% of contacts) and other (39% of contacts) account for significantly less time. If we partition contacts by their frequency, then we observe that subjects generally spend most time on a given day with people whom they meet regularly, although contacts met less often than once a week were the second highest contributors to total contact hours. Unsurprisingly, contacts that are met for an hour or more dominate the contact hours. Finally, we find that the total time spent with contacts decreases with distance from home, reflecting the shorter duration we spend at longer distances. In summary, these results strongly support the assertion that we generally catch infection from, and spread infection to, those individuals who we see frequently and for long durations; the large number of infrequent, short-duration contacts that we make each day may be largely epidemiologically irrelevant.

In a similar manner, we can examine how clustering (the presence of a transitive link in the previous week) varies with reported covariates; we have already seen that clustering is strongly dependent on the age of the respondent. We find the average clustering, considering all contacts, was 0.46, whereas the clustering between contacts who touch was higher (0.53). When we consider clustering within different contextual settings, home contacts have by far the highest level of clustering (0.70), whereas work contacts are also highly clustered (0.51); contacts in home and work settings are likely to encounter one another owing to the restricted environment in these settings. We find clustering increases with duration of contact and frequency of encounters, with the lowest values of clustering among individuals for whom respondents said they spent 10 min or less, or encountered for the first time on the day of the survey. Contacts which are met for long periods or with high frequency are more likely to meet each other than contacts which are brief or infrequent. This is possibly a reflection of the strong correlations between many of the contact properties; for example, home contacts are typically both of long duration and highly regular.

The result for distance is somewhat counterintuitive. Contacts made within two miles are dominated by home contacts and therefore have high clustering; however, the highest values of clustering occur for contacts made 50 miles or more away from home. We hypothesize that this may be due to differences in the purpose behind contacts made at longer distances compared with those made at shorter distances. For example, work contacts made at long distances may be generated through business meetings, where encounters are made within a highly clustered group. Similar societal factors may structure other types of contact at longer distances from home and the types of social interactions encountered owing to travelling longer distances.

To assess potential biases introduced through the different data collection methods, we considered clustering separately for online and postal surveys, including and excluding groups data, as well as all together (see electronic supplementary material, §4*b*). These show that clustering within groups is comparatively high as one might expect; however, including groups in the analysis increases the overall clustering by only 3 per cent. Online survey responses yielded lower clustering values than postal responses, despite higher within-group clustering, as more online respondents reported no transitive links and therefore 0 clustering (see electronic supplementary material, figure S5). We also tested the assumption that only some transitive links within groups were real, by scaling the number of within group links by 50 per cent and 75 per cent; for both assumptions, the clustering values remain high.

## 4. Discussion

Close-contact infections rely on the social contacts of suitable hosts for sustained transmission to occur; quantifying such encounters can provide explanatory insights for observed incidence patterns [22]. The only large-scale study conducted into social contacts, POLYMOD [16], has been influential in shaping the way that mathematical models of disease transmission are parametrized [23–27]. While the POLYMOD study measured many properties of encountering contacts, it did not measure other aspects of social networks which may be equally important in the spread of infection [2] or the impact of control [14,23]. This study represents, to the best of our knowledge, the largest survey of contact patterns conducted for a national population and includes metrics of social networks, such as clustering and distance from home, and characteristics of respondents (occupation and household location) not previously collected together.

Response rates for the postal survey were relatively high for a random mailshot without follow-up [17]. There were biases in the age and sex of respondents to our study: females were approximately twice as likely as males to participate, and younger age groups of both sexes (below 18) were under-represented. We found little bias in postal responses for different survey days of the week. One and two-person households are over-represented, and our analysis may not fully capture all aspects of within-household contacts. We believe the biases reflect the natural diversity in both health concerns and available time in the population; the anonymity of the study means that it is impossible to follow-up non-respondents to achieve a more even sample. Web-based survey methods have great potential to ease the burden of reporting complex egocentric data: our online survey removes many of the limitations imposed by space constraints of the postal questionnaire, and allowed a higher response from younger age groups. The pilot study of Beutels *et al*. [28] found little difference in the information collected via diary- and online-based questionnaires. In our larger study, although we found differences in demographic profiles of respondents between the two survey methods, within each demographic group, the two survey approaches produced similar results.

Our findings are, to some extent, dependent on the reliability of participants to interpret the questionnaire and describe interactions in a similar way. So far, no studies have validated the accuracy of contact diaries against other, more objective measures of social mixing; studies which have considered reciprocal agreement in the reporting of contact properties between participants found reciprocity increased with duration and intimacy of contact [15,29].

We find important differences when we compare our findings with previous work, with an average of around 27 contacts per day, more than twice that reported in [16] (11.74 for GB). Our survey has recorded some extremely large contact numbers, with a heavy-tailed distribution that influences the mean. We suggest that subtleties in the design of the questionnaire may significantly affect reporting rates: our design purposely reduced the reporting burden for large numbers of contacts via groups which potentially encouraged participation from individuals with many contacts. Additionally, censoring effects arising from paper questionnaire design may have limited previous studies to capture the right-hand tail of the distribution.

A power-law model is found to fit the tail of the contact degree distribution better than alternative distribution models [21]: we believe this is the first convincing evidence for power-law distributions in social encounter networks. However, from a pathogen perspective, there is limited potential associated with very high numbers of contacts; individual encounter time and therefore transmission opportunity per encounter must reduce as the number of contacts gets very high. Thus, although degree distribution may indeed be heavy-tailed, we hypothesize that the distribution of secondary cases generated by an infected individual displays far less variation [21,30] and is more reliably captured by total contact time.

The heterogeneity in number of contacts and total contact time was not randomly distributed but was strongly correlated with individual-level characteristics. As found in previous studies, age was a clear determining factor [16], with school children having the highest levels of contact while contact time decreased consistently from age 45 onwards. In addition, we found that certain occupations inherently have higher contact times and therefore greater potential for becoming infected as well as contributing more to onward transmission. Both children (who are typically highly susceptible to respiratory infections) and healthcare workers (who would be expected to provide front-line services during an epidemic) are among the groups with greatest potential exposure. We expect assortativity that may arise from interacting with others in the same occupation to amplify these effects and would also act to raise population-level measures such as the basic reproductive ratio (the number of secondary cases caused by a single infectious case in a totally susceptible population). There may be extra benefit in targeting these groups to reduce their epidemiological role and depress the spread of infections. We therefore conclude that understanding links between professions and their contact networks may provide a powerful tool with which to target prophylactic infection control.

To the best of our knowledge, this is the first study of clustering within personal social contact networks for a large random sample of individuals, and the first to measure clustering in conjunction with other participant and contact information. A much smaller study [15], using a convenience work-based peer group, found a clustering coefficient (unweighted by contact time) of 0.69, whereas our study found a work-based clustering coefficient of 0.51 (weighted) and 0.43 (unweighted), across a much broader range of occupations and demographics.

This study not only verifies the results of previous surveys about the importance of age structure, but also highlights four other epidemiologically important observations: first, that there is extreme heterogeneity in the number of social contacts although this heterogeneity is tempered if we consider the more applicable measure of total contact time; second, that for adults, occupation plays a role in determining the contact pattern and hence epidemiological risk; third, that there are high levels of clustering (transitive links) in many social settings which can dramatically alter predictions for infection spread and control; and finally, that there is a subtle interplay between the duration and frequency of contacts, and the distance travelled to make them. The quantification of these network parameters allows us for the first time to judge the relative risks for different elements of society and for different types of social interaction. We therefore believe that these findings form a basis for more realistic modelling studies in the near future and indicate heterogeneities that could be usefully targeted to improve infection control.

## Notes

Approval for this study was granted by the ethics committees of University of Warwick and the University of Liverpool.
